# Implementing Personalized Medicine in the Academic Health Center

**DOI:** 10.3390/jpm6030018

**Published:** 2016-09-21

**Authors:** Scott T. Weiss

**Affiliations:** Partners Center for Personalized Medicine, Partners Health Care, Boston, MA 02115, USA; scott.weiss@channing.harvard.edu; Tel.: +1-617-525-5136

## 1. Introduction: What is Partners Health Care?

Recently we at Partners Health Care had a series of articles in the Journal of Personalized Medicine describing how we are going about implementing Personalized Medicine in an academic health care system [1,2,3,4,5,6,7,8,9,10]. In this editorial I would like to review what we consider the essential features of our program and what we would consider the standards for success. Partners Health Care is the parent organization for Massachusetts General Hospital (MGH) and Brigham and Women’s Hospital (BWH). The health system has 68,000 employees and is the largest employer in the state of Massachusetts. Its two academic medical centers (MGH and BWH) are both ranked within the top ten by US News and World Report. Collectively, the two hospitals have the largest National Institutes of Health (NIH) funded research program of any academic medical center in the US at approximately 1.7 billion dollars/year.

## 2. Personalized vs. Precision Medicine, What is the Difference?

I do not believe that it matters all that much what term you use to describe what we are trying to do, but these terms have slightly different connotations and it is worth considering what they are. Personalized Medicine is the older term and carries the connotation of individualized treatment. It is misleading in two respects. First, older physicians have suggested that all medicine is personalized in that we are always trying to maximize the health of the person we are treating in all aspects of their care, not just in the application of genomics to their problem, but also in the realm of listening, and responding, to the patients’ social and psychological concerns. Personalized medicine also has the connotation that the application of genomics is at the individual and not the group level, and seems to not consider cost as a critical factor in its application. Alternatively, Precision Medicine suggests that we are applying the right drug to the right patient at the right time, and does not distinguish between whether that genomic application is at the level of a group of patients that have some common genomic characteristic, or at the level of individual patients; it is focused primarily on the genomic application, not on the psychosocial aspects of care. In either case, whatever term you are using, the goal must be to improve care and reduce costs, not just improve care alone. Without a consideration of costs and the cost-benefit of introducing Personalized Medicine solutions, medicine will be doomed. Health care already consumes a significant portion of our government spending, however it is marginally reimbursed, and thus we cannot let the application of half way technologies that are not truly cost effective overtake the health care system.

## 3. What are the Essential Features Needed to Implement Personalized Medicine?

There are five essential features that any academic health system must have if it intends to take advantage of the genomic revolution: it must have an electronic medical record system, it must be able to empower its researchers, it must be able to support clinicians, it must train researchers and clinicians, and it must only implement cost effective genomic practices across the health system. We will discuss each of these five features and how we have attempted to deal with them at Partners Health Care.

### 3.1. Electronic Medical Record System

Although all Partners hospitals had electronic medical records systems, they did not talk to each other. A critical decision by Partners leadership was made to implement a common electronic record system eCARE based on EPIC, across the whole system including all 12 Partners Hospitals and all of its clinical practices. A patient will now be able to enter any Partners facility and have his or her medical record accessed on site. Electronic medical records (EMR) are needed to deliver genomic content to practitioners. After a real push by the Obama administration, over 80% of all US health care practitioners nationally have such a system. Given the increasing volume of genomic data, such records will ensure higher uniformity of care, and a greater ability to monitor patient outcomes and deliver genomic data.

In addition to having a common EMR, the health system must be able to deliver genetic and genomic results from its molecular laboratories to clinicians. Genomic data has the potential to be large volume, complex, and changing. Every academic health system needs to have a software suite that will be able to keep up with this volume of data and change the data as new results become available. Partners Health Care has developed a software suite: GeneInsight, that directly links the systems molecular laboratories to clinicians for the delivery of these results through the electronic medical record system. GeneInsight has been sold to a commercial laboratory vendor, Sunquest Information Systems (Tucson, AZ, USA), and is now available to other Academic Medical Centers AMCs across the country, both assuring its viability but also longevity. Currently the system manages both somatic and germ line mutations but can be expanded to cover other genomic type data.

### 3.2. Empowering and Training Researchers

Partners Health Care has approximately 2200 researchers with NIH funded grant support and over 6000 who have some research component to their job. Part of Personalized Medicine is to empower these investigators to utilize genetic and genomic technologies to greater effectiveness to translate the promise of the human genome and multiple omics into clinical practice. To accomplish this, Partners Personalized Medicine has established a series of core services that investigators can utilize for minimal fees to enhance their research efforts. These services include a Translational Genomics Core for sequencing of DNA and RNA [9], a Biobank of over 47,000 Partners patients with serum, plasma, and DNA [6] linked to the research version of the electronic medical record called the RPDR [7], and a Laboratory of Molecular Medicine that performs germ line genetic and genomic testing [5]. The three core laboratories are linked via a common laboratory information management system [3] and are linked to investigators via a research portal that brings all clinical data (imaging, EMR, clinical labs, etc.) into the research realm [7]. Additional data has also been generated and provided to researchers for free. For example, all Biobank participants are being genotyped using the Illumina MEGA chip (Illumina, San Diego, CA, USA) and this GWAS (Genome-wide association study) data is given to investigators for free. Computationally, a phenotyping core uses machine learning algorithms and natural language processing to create high quality disease phenotype data from the EMR [4]. These tools are designed to give Partners investigators tools to compete effectively for grants in the genomic era and advance personalized medicine into clinical practice.

### 3.3. Empowering and Training Clinicians

We have already mentioned the single biggest system wide factor for empowering clinicians and that is the GeneInsight software. However, this system is only effective if genetic tests are performed by the system’s own molecular diagnostic laboratories and are thus directly entered into the EMR. Tests that are completed as send outs are placed in the record but not in a way that they can be easily utilized to monitor care or used for research. The other major factor faced by clinicians is that most molecular diagnostics are marginally reimbursed and hence have the potential to be billed directly to patients. This is a huge negative aspect for clinicians as they do not want patients to incur these additional costs, nor do they have the time to file onerous paper work seeking preapproval for genetic testing from third party providers. As a health system, we are currently evaluating all aspects of our genetic testing program in an attempt to maintain high quality as well as reduce cost and maintain our focus on diagnostic excellence. Another area of ongoing activity is to enhance development of genetic testing and improve our molecular genetic test offerings for clinicians particularly in the form of support for novel clinical service lines. Another area of innovation is the development of health applications (apps) that can help clinicians manage complex, difficult, and costly patients (e.g., heart failure, renal failure). These apps will integrate EMR, genomic, and other data to aid clinician decision making and hopefully reduce costs and improve care. Finally, it is not all about genetics, but also about genomics and systems medicine and the integration of genetic and genomic data with other health data such as imaging, and other laboratory data to improve clinical decision making and health outcomes.

### 3.4. Training of Researchers

Participation in federally funded research in translational genomics has significant benefits for the academic health center. It trains future staff, provides access to the latest translational activities and best practices, and places the health center at the center of innovation in health care. Partners Personalized Medicine participates in: Electronic Medical Records and Genomics (eMERGE), Clinical Genome Resource (CLINGEN), Clinical Sequencing Exploratory Research (CSER), and other translational activities sponsored by the NIH, with this effort designed to both gain new knowledge that can be applied to care but to also train the next generation of researchers. Use of the Partners HealthCare Personalized Medicine (PPM) infrastructure has facilitated the development of these competitive grant proposals. Institutional training grants from the NIH have also facilitated this activity [10].

### 3.5. Cost Effectiveness

Health care is a very low margin business where reimbursement from the government or third party payers is required for services rendered. These payers attempt to control costs by denying payment for services that have not been proven to be cost saving or critical to care. Molecular diagnostics are rarely if ever critical to care decisions, and where they have been shown to be, the jury is still out on their ultimate value [8]. We are clearly not ready for whole genome sequencing on everyone and making that the standard of care. Many believe that sequencing either the germ line or somatic line alone will not be enough to ultimately advance personalized medicine. Regardless of where you stand on the continuum of enthusiasm for whole genome sequencing, we clearly need to be implementing those personalized medicine solutions that are both critical to high quality care and cost saving. Demonstration of the latter is critical to convince payers of the value of personalized medicine solutions. At Partners we strive not just for Personalized Medicine breakthroughs but we strive to implement those of greatest value to our patients.
